# Chemical and Technological Characterization of Dairy Products

**DOI:** 10.3390/foods9101475

**Published:** 2020-10-16

**Authors:** Michele Faccia

**Affiliations:** Department of Soil, Plant and Food Sciences, University of Bari, Via Amendola 165/A, 70126 Bari, Italy; Michele.faccia@uniba.it

The dairy sector is facing a decisive challenge in developed countries, which could deeply influence its future and its historical status of being a pillar for human nutrition. The most challenging issue is to give suitable answers to the demand for nutritionally balanced and environmentally sustainable products, the two main aspects of the new “food paradigm” that increasingly sees foods as drugs (specifically renamed as “nutraceuticals”) and imposes a stringent eco-friendly approach in their production (“green foods”) [1,2]. In this context, all animal products are often met with hostility that is not always justified under the scientific point of view, particularly when it results in imposing their complete elimination [3,4]. As expected, this challenge has soon been met by researchers in dairy and food sciences, who are giving suitable scientific answers and are driving dairy farms and industries to develop new products and processes that can better satisfy the new requirements. In this context, the Special Issue “Chemical and Technological Characterization of Dairy Products” has collected 13 articles (12 original researches and one review) that give an interesting contribution to the field. The articles can be grouped into three categories: the first one concerns product innovation and includes eight papers reporting technological and compositional details on innovative dairy products developed in line with the nutritional and/or environmental requirements mentioned above; the second one has an interdisciplinary approach (animal husbandry-food technology) and is made of three studies aimed to deepen the influence of the cattle rearing conditions on cheese quality; the third one contains two papers dealing with different aspects of dairy science.

*Product Innovation*. Abdel-Hamid et al. [5] and Dhawi et al. [6] developed different types of functional buffalo milk yogurt and investigated their chemical, microbiological, organoleptic and bioactivity characteristics. In the first case, a functional yogurt was obtained by fortifying milk with Chinese sweet tea extract (*Rubus suavissimus* S. Lee leaves). The phenolic compounds included as a consequence of extract addition improved biological activity in terms of antioxidant and antihypertensive activity and inhibition of the Caco-2 carcinoma cell line; on the other hand, the viability of the yogurt starter cultures during refrigerated storage was not significantly affected. Finally, the sensory analysis demonstrated a high acceptability of the product and allowed for establishing the most suitable level of fortification. In the second paper, two different plant-based ingredients were used as yogurt-fortifying agents: fenugreek (*Trigonella foenum-graecum*) seed flour and *Moringa oleifera* seed flour. *Moringa oleifera* samples had higher values of phenolic compounds and antioxidant activity as compared to fenugreek, and exerted higher antibacterial activity against several undesired species. On the contrary, the viability of *Streptococcus thermophilus* and *Lactobacillus bulgaricus* was improved. Incorporation of the flours caused a modification of the concentration of mineral compounds, with connected increase of some valuable elements, and of the sensory characteristics. Saleh et al. [7] investigated the use of different types of starch (corn, sweet potato, potato, Turkish beans, and chickpea) as fat replacer in set yogurt. The results show that all starches used reduced syneresis and improved firmness of the product, but at different level due to their origin and amylose content. Yogurts with corn and tuber starches had the highest viscosity, chickpea starch exhibited the highest storage moduli. Different starches behave differently during yogurt storage, and some of them performed better at the beginning of the storage period. Sensory evaluation showed a preference for starch-containing samples as compared to the control, regardless of the starch type. The work of Chen et al. [8] focused on a stirred acidified dairy gel used as a model system for studying ingredient functionalities in yogurt. The study regarded the effect of including black tea infusion on the physicochemical properties, antioxidant capacity and microstructure of the system during a 28-day cold storage period. The results suggest that tea improved antioxidant capacity but significantly altered the texture of gel. The papers published by Serra et al. [9] and Faccia et al. [10] dealt with two types of innovative fresh cheeses. The first one regarded a cheese obtained both by sheep and buffalo milk by using kiwi juice as a coagulant, and it was addresses to assess the influence on flavor and on the presence of nutraceutical substances in comparison with calf rennet. Although the kiwifruit extract caused a longer coagulation and syneresis time than calf rennet, a positive effect on the nutraceutical properties of the cheese was found due to a higher presence of polyphenols and phytosterols. Contrastingly, the profile of volatile organic compounds was not deeply affected, since the typical odorants of the kiwi aroma were poorly transferred to the cheese; the authors suggested the need for further study to evaluate the impact on the sensory characteristics. Faccia et al. described the production technology and the compositional/sensory characteristics of a cheese obtained by the enzymatic coagulation (microbial rennet) of donkey milk. Donkey milk coagulated rapidly, but the curd remained soft, and was only suitable for making fresh cheese; contrastingly, cow milk (used as control under the same conditions) coagulated almost instantaneously and gave rise to a semi-hard curd. Higher yields than those reported in the literature were obtained, probably due to the high protein content of the milk used. The main compositional and sensory characteristics of the cheese were assessed and discussed. Solid Phase Micro Extraction-Gas Chromatography-Mass Spectrometry (SPME-GC-MS) analysis allowed for identifying 11 volatile compounds in milk and 18 in cheese. The other two papers included in this category regard two very innovative products. Buhler et al. [11] investigated the effect of defatting cheese (instead of milk) as an alternative way for obtaining a low-fat Parmigiano Reggiano cheese type. Two defatting procedures were tested, and the composition of the nitrogen fraction of the obtained products were compared. Moreover, the nitrogen compounds were extracted and subjected to simulated gastrointestinal digestion in order to test the antioxidant and angiotensin converting enzyme (ACE) inhibition capacities of the digests. The results obtained show that the defatted products kept the same nutritional properties of the whole cheese. Tian et al. [12] made use of the emulsifying property of polymerized goat whey proteins (PGWP) to prepare soy isoflavones (SIF) nanoparticles. High encapsulation efficiencies were ascertained, and the inclusion of isoflavones increased the particle size and lower zeta potential compared with PGWP alone. The secondary structure of the proteins changed after interacting with SIF, with the transformation of α-helix and β-sheet to disordered structures. The authors concluded that PGWP might be a good carrier material for the delivery of SIF in functional foods.

*The influence of rearing conditions on cheese quality*. Formaggioni et al. [13] compared the fatty acid profile and the sensory properties of a traditional cheese manufactured from milks of cows reared and fed under different conditions. The experimentation demonstrated that the fat of the cheese obtained from cows fed indoors was richer in medium-chain fatty acids, whereas grazing positively influenced the concentrations of long-chain and unsaturated fatty acids, such as oleic, Conjugated Linoleic Acids (CLAs) and omega 3 fatty acids. Nevertheless, the sensory analysis showed that the tasters were not always able to find significant differences among the cheese samples. Martino et al. [14] dealt with the relationships between feeding dairy sheep with zinc (a key mineral that is not stored in the animal body) supplementation and cheese characteristics. The feeding strategy induced a significant increase in zinc concentration in milk, but it also seemed to be connected to an increase in vaccenic, rumenic and total polyunsaturated fatty acids, in both milk and cheese. The study suggests possible positive effects of dietary zinc supplementation in improving the nutritional characteristics of fresh and ripened dairy products. Franceschi et al. [15] deepened the effect of season and factory on cheese-making efficiency in Parmigiano Reggiano cheese manufacture. They focused particularly on the relationship between cheese-making losses and protein and fat content by considering the production process of 288 Parmigiano Reggiano cheese moulds manufactured in three different cheese factories. The authors reported that estimated cheese losses strictly depend on milk characteristics, in particular, milk fat, casein contents, and rennet coagulation properties; they concluded that estimated cheese-making losses of protein and fat can be used as an instrument for controlling the manufacturing process.

Two further papers complete the Special Issue: an Original Article and a Review Article. The first one [16] is a very interesting study in which confocal Raman microscopy was applied to investigate the microstructure of high moisture cream cheese after freezing and thawing in comparison with confocal laser scanning microscopy. Raman spectroscopy is very interesting since it allows for observing different classes of molecules in situ, in complex food matrices, without modifying them. The results show that it was possible to identify and map the large water domains formed during freezing and thawing and that this technique could be complementary to confocal laser scanning microscopy. In addition, the microscopy data complemented the information derived from low-resolution Nuclear Magnetic Resonance (NMR), suggesting that NMR and Raman microscopy can be complementary to distinguish between different commercial formulations, and different destabilization levels. Finally, the Review by Manoni et al. [17] supplies exhaustive information about the formation of bovine milk fat globules and the milk fat globule membrane (MFGM), highlighting the main similarities and differences across the MFGM proteomes of the most-studied mammal species. Moreover, the potential supplementation of MFGM fractions in infant formula in order to underline the beneficial effects exerted by MFGM bioactive components was investigated.

In summary, the Special Issue “Chemical and Technological Characterization of Dairy Products” offers readers a series of innovative information that can be useful both for developing new research ideas and for developing new types of dairy products.
